# CYP3A4 inhibitors may influence the quantification of [^123^I]I-FP-CIT SPECT scans

**DOI:** 10.1007/s00259-024-06748-0

**Published:** 2024-05-11

**Authors:** Jan Booij, Eda Yağci, Zulfiqar H Sheikh, Youssef Chahid

**Affiliations:** 1 Department of Radiology and Nuclear Medicine, University of Amsterdam, Amsterdam UMC, Amsterdam, The Netherlands; 2 Department of Pharmacy, University of Amsterdam, Amsterdam UMC, Amsterdam, The Netherlands; 3 Pharmaceutical Diagnostics, GE Healthcare, Chalfont Saint Giles, Nightingales Ln, UK; 4 Department of Radiology and Nuclear Medicine, University of Amsterdam, Amsterdam UMC, Meibergdreef 9, Amsterdam, 1105 AZ The Netherlands

**Keywords:** [^123^I]I-FP-CIT, DaTSCAN, Dopamine transporter imaging, Drug interactions, CYP3A4 inhibitors

## Abstract

**Purpose:**

[^123^I]I-FP-CIT SPECT is an imaging tool to support the diagnosis of parkinsonian syndromes characterized by nigrostriatal dopaminergic degeneration. After intravenous injection, [^123^I]I-FP-CIT is metabolized for a small part by the enzyme CYP3A4, leading to the formation of [^123^I]I-nor-β-CIT. [^123^I]I-nor-β-CIT passes the blood-brain barrier and has a very high affinity for the serotonin transporter (SERT). The SERT is expressed in the striatum and cortical areas. So, at least theoretical, the use of frequently used CYP3A4 inhibitors (like amiodarone) may influence the specific to non-specific striatal [^123^I]I-FP-CIT ratio. Here we tested this novel hypothesis.

**Methods:**

Using a retrospective design, we determined the specific to non-specific striatal [^123^I]I-FP-CIT ratio (using BRASS software) in 6 subjects that were using an CYP3A4 inhibitor and 18 matched controls. Only subjects were included with a normal rated [^123^I]I-FP-CIT SPECT scan, and all participants were scanned on the same brain-dedicated SPECT system.

**Results:**

The specific to non-specific (assessed in the occipital cortex) striatal [^123^I]I-FP-CIT binding ratio was significantly higher in CYP3A4 users than in the control group (3.52 ± 0.33 vs. 2.90 ± 0.78, *p* < 0.001).

**Conclusion:**

Our preliminary data suggest that the use of CYP3A4 inhibitors may influence striatal [^123^I]I-FP-CIT binding ratios. This information, when reproduced in larger studies, may be relevant for studies in which quantification of [^123^I]I-FP-CIT SPECT imaging is used for diagnostic or research purposes.

## Introduction

[^123^I]I-FP-CIT SPECT is a well-validated imaging tool to investigate the integrity of the dopaminergic nigrostriatal pathway in parkinsonian patients by targeting the dopamine transporter (DAT) [1]. In routine practice, analysis of [^123^I]I-FP-CIT SPECT scans typically involves a visual read. However, in many centers, a semi-quantitative approach is added to the visual read to classify the scan as either normal or abnormal.

Recently, we published a systematic review on the potential effects of medication on striatal [^123^I]I-FP-CIT binding. As expected, medications that target the DAT, such as methylphenidate, were identified as potentially influencing the interpretation of [^123^I]I-FP-CIT SPECT scans [2]. Interestingly, we found no studies on medications that may influence striatal [^123^I]I-FP-CIT quantification due to its potential influence on the metabolism of this radiotracer.

**Fig. 1:**
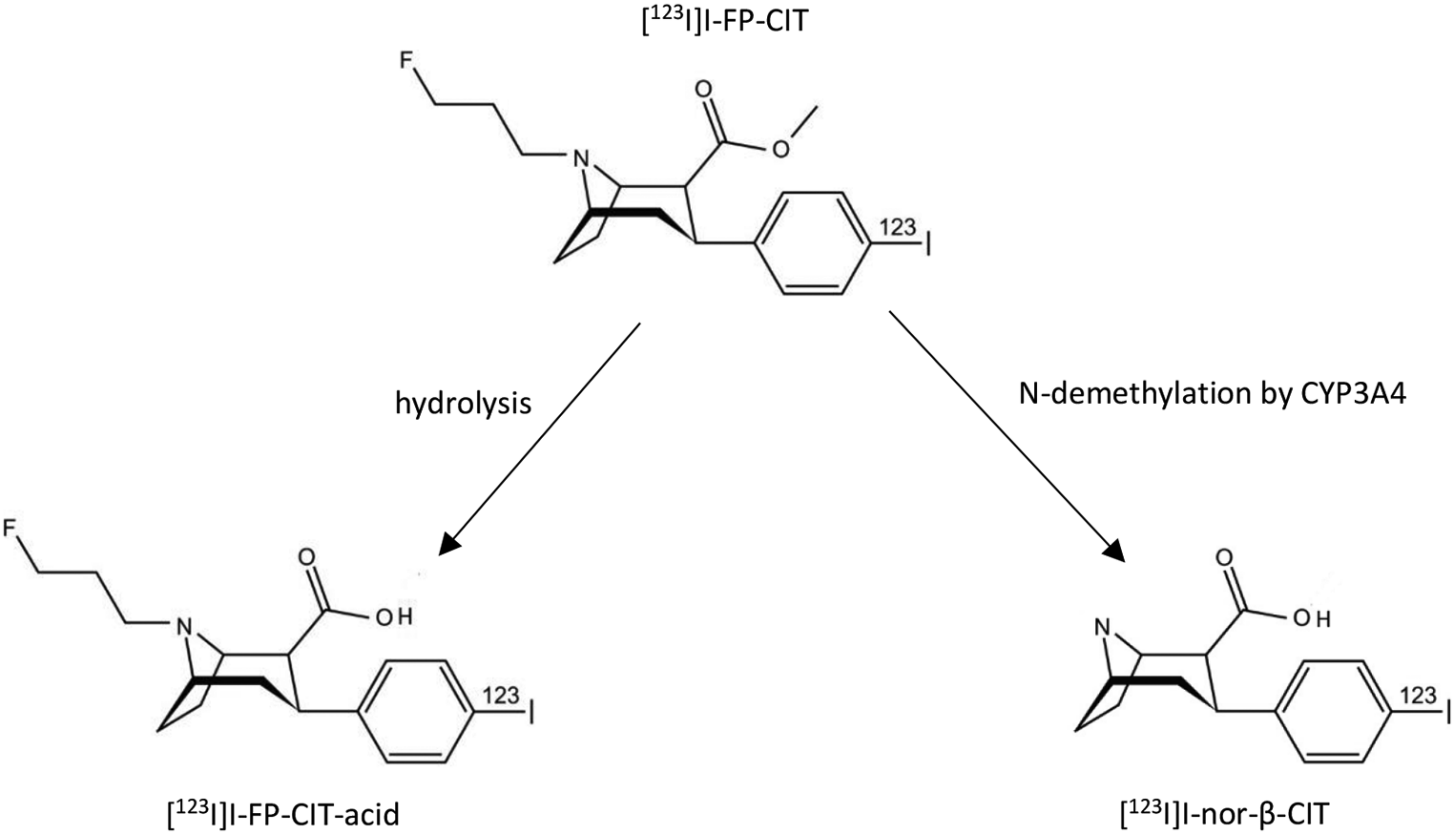
The metabolism of [^123^I]I-FP-CIT after intravenous injection. The main metabolic pathway involves the hydrolysis of the ester group. In this process, the ester bond is cleaved, resulting in the formation of [^123^I]I-FP-CIT acid. This metabolite is likely not lipophilic enough to cross the blood-brain barrier (BBB). Another metabolic route involves N-demethylation, catalyzed by the enzyme CYP3A4, leading to the formation of a small amount of lipophilic [^123^I]I-nor-β-CIT, which can pass the BBB, and binds to both the serotonin and dopamine transporter. This Figure is largely reproduced from Fig. 2 [3]

Like all drugs, [^123^I]I-FP-CIT is metabolized in the human body after administration [3]. The main metabolic pathway involves the hydrolysis of the ester group, resulting in the formation of [^123^I]I-FP-CIT acid (Fig. 1) [4]. However, this metabolite is likely not lipophilic enough to cross the blood-brain barrier (BBB) [5]. Another metabolic route involves N-demethylation, catalyzed by the enzyme CYP3A4, leading to the formation of lipophilic [^123^I]I-nor-β-CIT (Fig. 1) [5]. This metabolite is a radiotracer formed in a small amount (< 4% at 1 h postinjection, but may be > 10% at 3 h postinjection) which can cross the BBB [5]. Thus, so-called CYP3A4 inhibitors (like amiodarone) are at least theoretically able to (partly) block the formation of [^123^I]I-nor-β-CIT in subjects injected with [^123^I]I-FP-CIT. Importantly, [^123^I]I-nor-β-CIT binds with a very high affinity to the serotonin transporter (SERT), but has also a high affinity to the DAT [6].

Interestingly, we and other showed that [^123^I]I-FP-CIT is not a selective DAT radiotracer, but has also modest affinity for the SERT [7, 8]. [^123^I]I-FP-CIT SPECT scans are commonly quantified by determining the striatal binding versus non-specific binding. Often, binding in the occipital cortex is used to assess the amount of non-specific binding. However, both the striatum and occipital cortex are not devoid of SERTs. Indeed, we and others showed that striatal [^123^I]I-FP-CIT binding ratios can be influenced by the use of selective serotonin reuptake inhibitors [7, 8].

To our knowledge, we now examined for the first time the potential effects of CYP3A4 inhibitors on the quantification of [^123^I]I-FP-CIT SPECT scans. Based on the above described metabolism of [^123^I]I-FP-CIT, and taking into account that a change in the denominator of a ratio has a larger impact on the calculated ratio than a similar change in the numerator [7], we postulate that the use of CYP3A4 inhibitors may increase the striatal [^123^I]I-FP-CIT binding ratio, particularly by reducing the non-specific binding due to the diminished formation of [^123^I]I-nor-β-CIT.

## Materials and methods

### Subjects

The study was retrospectively conducted using data obtained for clinical purposes at the Amsterdam University Medical Centers (Amsterdam UMC), University of Amsterdam, the Netherlands. Ethical approval was waived by the local Ethics Committee. The study adhered to the principles of the 1964 Helsinki Declaration and its subsequent amendments. Patients were eligible to be included if they had received an [^123^I]I-FP-CIT SPECT scan for diagnostic purposes during the period from January 2016 until October 2023 and were using an CYP3A4 inhibitor. Well known CYP3A4 inhibitors are medications like amiodarone and diltiazem [9]. Patients data were collected from EPIC (electronic patient file). Patients with an abnormal rated [^123^I]I-FP-CIT scan were excluded, since many factors (such as disease duration) may influence the striatal binding ratio in patients with neurodegenerative diseases like Parkinson’s disease [1]. Additionally, patients with unknown medication information and patients taking medications known to decrease the striatal binding of [^123^I]I-FP-CIT were also excluded (2,3). Subsequently, we selected subjects who were not using CYP3A4 inhibitors for the control group, which was three times larger than the case group. We matched the control cases for age, sex, body mass index (BMI), smoking, alcohol and drug use, and the amount of administered radioactivity.

### [^123^I]I-FP-CIT SPECT scans

All subjects were injected with approximately 111 MBq [^123^I]I-FP-CIT, and scanned 3 h later. Details of the brain-dedicated InSPira system, acquisition protocol, attenuation correction, and reconstruction algorithm have been described earlier [10]. The SPECT scans were quantified automatically using the Brain Registration and Analysis Software Suite (BRASS; HERMES Medical, Sweden), as earlier described (10). The specific striatal to non-specific binding (assessed in the occipital cortex) ratios were assessed for the whole striatum, and its subregions, the caudate nucleus and putamen. We also assessed the activity separately in the striatum and occipital cortex (mean counts/voxel), corrected for body weight and injected dose.

The main outcome was the difference of specific to non-specific striatal binding ratio (mean left and right sides) between the two groups under study. In an exploratory analysis, we also examined differences between the 2 groups for binding ratios for the caudate nucleus and putamen, and the total activity in the striatum and occipital cortex.

### Statistical analysis

To compare groups as appropriate, we used different tests. The unpaired T-test was used for continuous variables, with data presented as mean ± standard deviation. Categorical variables were analyzed using Fisher’s exact test, and results are presented as counts and percentages (%). Statistical significance was defined as *P* < 0.05.

## Results

Both groups were similar regarding age, sex, BMI, and use of drugs. In the case group (*n* = 6), 5 patients were using the CYP3A4 inhibitor amiodarone, and 1 patient diltiazem (Table 1). No significant differences were observed in the injected activity of [^123^I]I-FP-CIT.

**Table 1:** Patient characteristics of the study population

Characteristic	Use of CYP3A4 inhibitor		*P*-value
	Yes (*n* = 6)	No (*n* = 18)	
**Sex**			0.649 *
Male	2 (33.3)	9 (50.0)	
Female	4 (66.7)	9 (50.0)	
**Age (years)** †	73.0 ± 12.0	70.5 ± 7.5	0.649 #
**BMI (kg/m**^**2**^**)** †	25.1 ± 6.6	25.5 ± 4.0	0.907 #
**Smoking**			0.562 *
Yes	0 (0)	3 (16.7)	
No	6 (100)	14 (77.8)	
Unknown	0 (0)	1 (5.6)	
**Alcohol use**			0.404 *
Yes	3 (50)	7 (38.9)	
No	3 (50)	9 (50.0)	
Unknown	0 (0)	2 (11.1)	
**Drugs use**			0.732 *
Yes	0 (0)	0 (0)	
No	5 (83.3)	13 (72.2)	
Unknown	1 (16.7)	5 (27.8)	
**Activity (MBq/kg)** †	1.67 ± 0.47	1.59 ± 0.33	0.697 #
**CYP3A4 inhibitors**			NA
Amiodarone	5	NA	
Diltiazem	1	NA	

† Mean ± standard deviation.

# Unpaired t-test.

* Fisher’s exact test.

NA = not applicable.

**Fig. 2:**
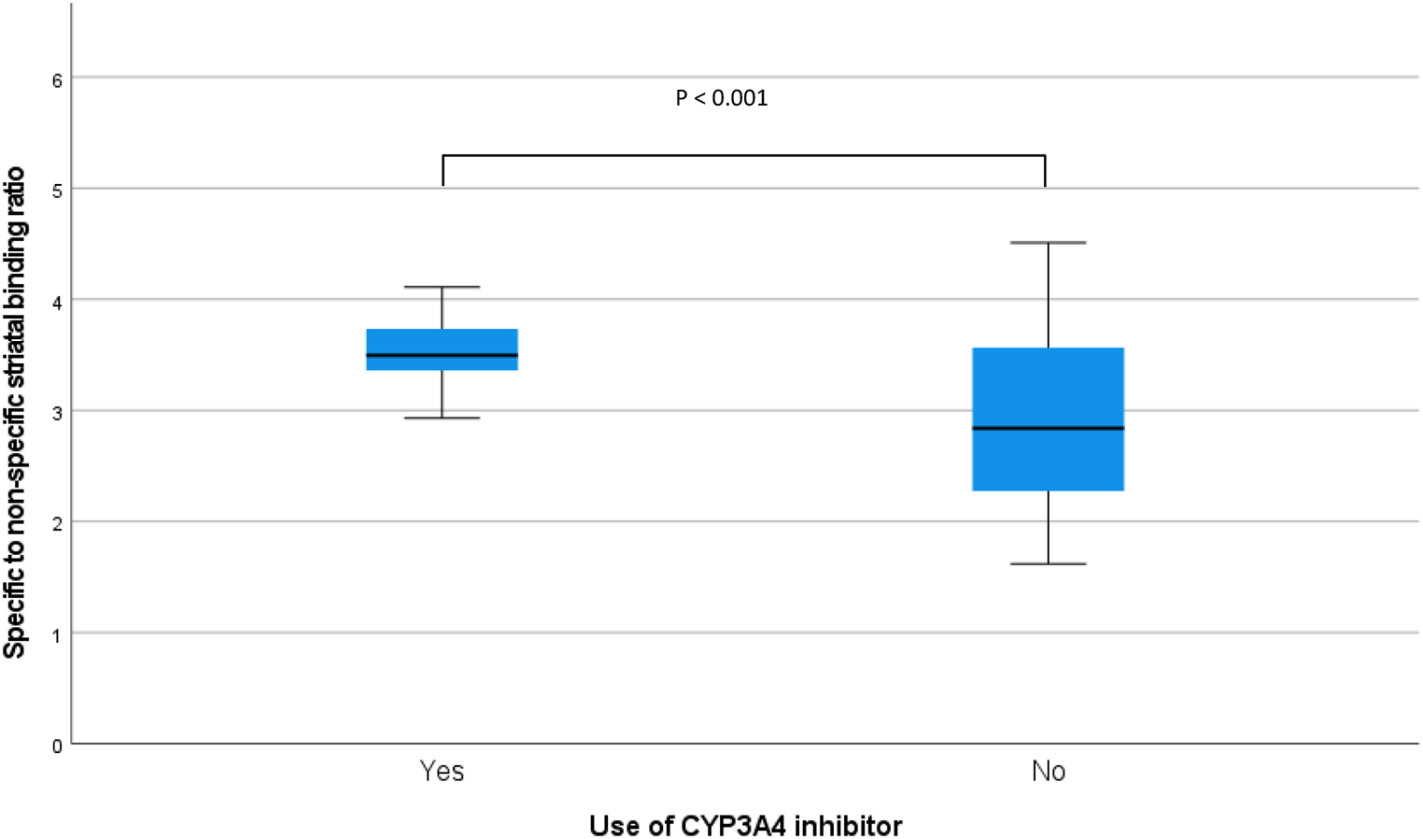
Specific to non-specific [^123^I]I-FP-CIT binding ratios for the whole striatum (mean left and right sides) for the control group (*n* = 18) and the 6 cases which were using an CYP3A4 inhibitor

The specific to non-specific striatal [^123^I]I-FP-CIT binding ratio was significantly higher in the case compared to the control group (3.52 ± 0.33 vs. 2.90 ± 0.78, *p* < 0.001; Fig. 2). The exploratory analyses showed that this was also true for the binding ratios of the caudate nucleus and putamen. Furthermore, while the counts in the occipital cortex were comparable between groups, the striatal counts were significantly higher (*p* < 0.001) in the cases than in the control group (Table 2).

**Table 2:** Specific to non-specific [^123^I]FP-CIT binding ratios (mean both sides) and measured activity in the striatum (mean both sides) and occipital cortex (representing non-specific binding)

Characteristics	Use of CYP3A4 inhibitor		*P*-value #
	Yes (*n* = 6)	No (*n* = 18)	
Specific to non-specific striatal binding ratio	3.52 ± 0.33	2.90 ± 0.78	< 0.001
Specific to non-specific caudate binding ratio	3.68 ± 0.30	3.04 ± 0.81	< 0.001
Specific to non-specific putamen binding ratio	3.34 ± 0.42	2.75 ± 0.78	0.002
Activity striatum*	544.83 ± 27.58	470.17 ± 94.87	< 0.001
Activity occipital cortex*	133.97 ± 12.05	123.78 ± 9.16	0.099

# Unpaired t-test.

*Activity in counts/voxel, corrected for injected dose and bodyweight.

## Discussion

Here we show, to our knowledge for the first time, preliminary data that the use of CYP3A4 inhibitors may influence striatal [^123^I]I-FP-CIT binding ratios.

It is reported that a small amount of [^123^I]I-nor-β-CIT will be formed by the enzyme CYP3A4 after [^123^I]I-FP-CIT administration [5]. Since [^123^I]I-nor-β-CIT can pass the BBB and has a very high affinity for the SERT and also a high affinity for the DAT, we postulated that the use of CYP3A4 inhibitors may influence striatal [^123^I]I-FP-CIT binding ratios [11]. On the one hand, our findings indeed supported this hypothesis. On the other hand, we postulated that lower formation of [^123^I]I-nor-β-CIT, due to the use of CYP3A4 inhibitors, may induce lower non-specific binding, which was not supported by our present data. In fact, the occipital counts were comparable between both groups, but the striatal counts were statistically higher in the group using CYP3A4 inhibitors than in the control group. It is well known that SERTs are expressed throughout the body [7]. So, it is possible that the use of CYP3A4 inhibitors will increase the availability of *parent* [^123^I]I-FP-CIT to enter the brain, due to lower formation of [^123^I]I-nor-β-CIT. This may lead to higher striatal [^123^I]I-FP-CIT binding ratios in CYP3A4 inhibitors users. Given the variability in the activity of CYP3A4 enzymes by up to a factor of 100 between individuals [12], it is expected that the use of CYP3A4 inhibitors will make the formation of [^123^I]I-nor-β-CIT more uniform. These more consistently lower levels of [^123^I]I-nor-β-CIT can explain the relatively narrow SD of the binding ratios in CYP3A4 inhibitor users (Fig. 2). Consequently, this may reduce the variability in the interindividual striatal [^123^I]I-FP-CIT to nonspecific binding ratio. These postulates can be tested in future studies.

**Fig. 3:**
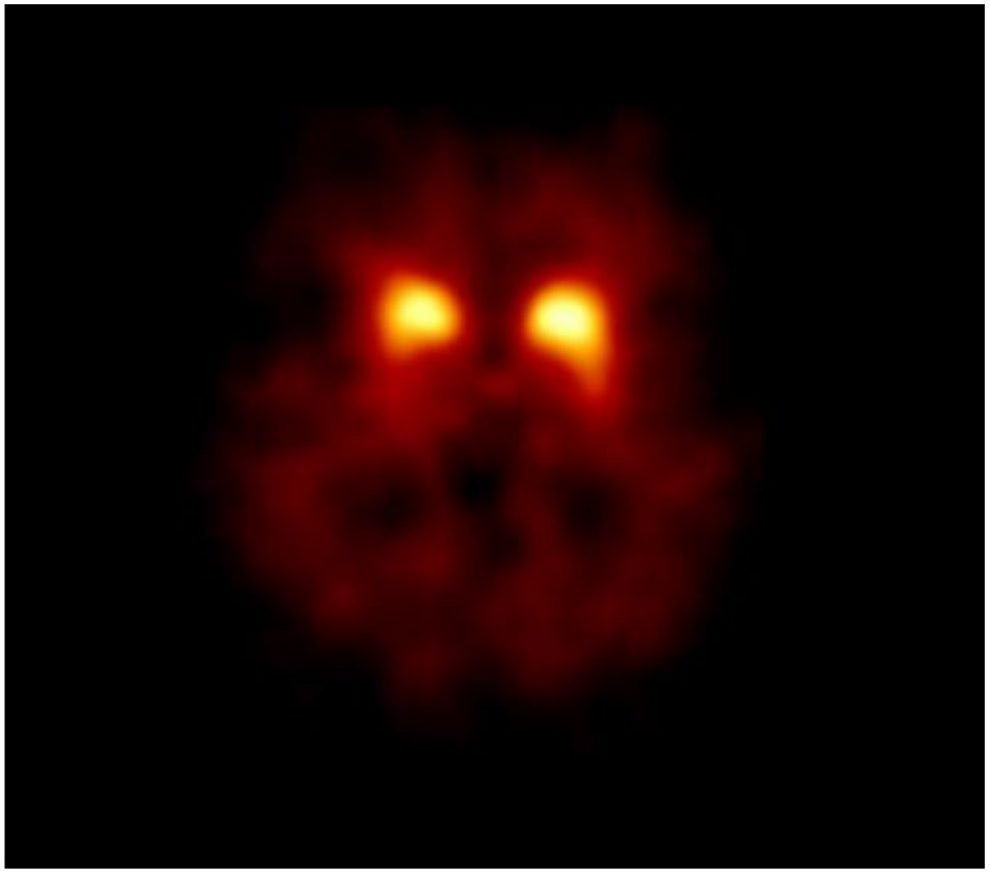
An [^123^I]I-FP-CIT SPECT scan performed in a patient suffering from Parkinson’s disease (PD), imaged while using an CYP3A4 inhibitor. Transversal slide at the level of the striatum. Please note the asymmetric striatal binding, and lower binding in the putamen than in the caudate nucleus. This pattern is typical of PD. This scan was rated as being abnormal based on the visual analysis as well as the quantitative analysis performed in BRASS, which showed decreased age-corrected binding ratios for the putamen bilaterally (data not shown)

In a recent systematic review, we examined medications that may reduce striatal [^123^I]I-FP-CIT binding ratios, and as such may induce false-positive cases [2]. However, our present data do not suggest that use of CYP3A4 inhibitors may induce lower binding ratios. If this finding is confirmed in future larger studies, then it is unlikely that the use of CYP3A4 inhibitors will lead to false-positive results. For illustration purposes, see a PD case who underwent [^123^I]I-FP-CIT SPECT imaging while using an CYP3A4 inhibitor (Fig. 3). Thus, a false-positive result will not be likely when a visual analysis is performed, also because it is unlikely that asymmetry and putamen-to-caudate ratios will be influenced by CYP3A4 inhibitors. Nevertheless, we cannot exclude that use of this class of drugs may increase the striatal binding ratios, e.g., in an early PD case, to borderline levels. Future studies may address this relevant topic.

Our present study has several limitations. Due to its retrospective design, we had to rely on the accuracy of the digitally reported data, introducing uncertainty about whether all included CYP3A4 users were indeed on this drug during DAT imaging. Nevertheless, it is unlikely that they were not using this medication, especially given that drugs like amiodarone and diltiazem are chronically used for severe clinical conditions such as heart arrhythmias. Additionally, in routine practice we do not advice to withdrawn these medications before DAT imaging. Furthermore, we had to rely on a small cross-sectional design. In future studies it may be of interest to perform a larger placebo-controlled study. Alternatively, a study can be designed in which the first [^123^I]I-FP-CIT SPECT scan is acquired just before starting the use of an CYP3A4 inhibitor, while a second scan will be acquired several weeks, in which the drug is taken under supervision at a standardized time before tracer injection. Furthermore, it may be relevant to examine the metabolism of [^123^I]I-FP-CIT during both imaging sessions. Also, in the present study we determined the non-specific binding in the occipital cortex. This is clinically relevant since many centers use commercially available software to quantify [^123^I]I-FP-CIT SPECT scans in routine practice. When using software such as BRASS and DaTQUANT, the occipital cortex is commonly used to assess the non-specific binding. However, to gain a better understanding of how CYP3A4 inhibitors may influence [^123^I]I-FP-CIT binding, it may also be of interest to evaluate binding in the ventral striatum, and non-specific binding in the inferior parts of the cerebellum, as these brain areas are devoid of DAT and SERT expression [7, 8]. Finally, here we focused on CYP3A4 inhibitors. In future studies it may be of interest to evaluate also the potential effects of CYP3A4 inducers on the striatal [^123^I]I-FP-CIT binding ratios.

In conclusion, our preliminary retrospective data suggest that the use of CYP3A4 inhibitors may influence striatal [^123^I]I-FP-CIT binding ratios. This finding may initiate larger prospective studies to reproduce our present novel finding, and to examine the mechanism by which CYP3A4 inhibitors may influence the quantification of [^123^I]I-FP-CIT SPECT scans.

## Data Availability

All data are available from the corresponding author on reasonable request.
